# Pregnancy in Women With Monogenic Diabetes due to Pathogenic Variants of the Glucokinase Gene: Lessons and Challenges

**DOI:** 10.3389/fendo.2021.802423

**Published:** 2022-01-05

**Authors:** José Timsit, Cécile Ciangura, Danièle Dubois-Laforgue, Cécile Saint-Martin, Christine Bellanne-Chantelot

**Affiliations:** ^1^ Department of Diabetology, Université de Paris, AP-HP, Cochin-Port-Royal Hospital, DMU ENDROMED, Paris, France; ^2^ PRISIS National Reference Center for Rare Diseases of Insulin Secretion and Insulin Sensitivity, Department of Endocrinology, Diabetology and Reproductive Endocrinology, Assistance Publique-Hôpitaux de Paris, Saint-Antoine University Hospital, Paris, France; ^3^ Monogenic Diabetes Study Group of the Société Francophone du Diabète, Paris, France; ^4^ Department of Diabetology, Sorbonne Université, AP-HP, Pitié-Salpêtrière Hospital, Paris, France; ^5^ INSERM U1016, Cochin Hospital, Paris, France; ^6^ Department of Medical Genetics, Sorbonne Université, AP-HP, Pitié-Salpêtrière Hospital, DMU BioGeM, Paris, France

**Keywords:** glucokinase, *GCK*-MODY, pregnancy, macrosomia, genotype, non-invasive fetal genotyping, insulin therapy

## Abstract

Heterozygous loss-of-function variants of the glucokinase (*GCK*) gene are responsible for a subtype of maturity-onset diabetes of the young (MODY). *GCK*-MODY is characterized by a mild hyperglycemia, mainly due to a higher blood glucose threshold for insulin secretion, and an up-regulated glucose counterregulation. *GCK*-MODY patients are asymptomatic, are not exposed to diabetes long-term complications, and do not require treatment. The diagnosis of *GCK*-MODY is made on the discovery of hyperglycemia by systematic screening, or by family screening. The situation is peculiar in *GCK*-MODY women during pregnancy for three reasons: 1. the degree of maternal hyperglycemia is sufficient to induce pregnancy adverse outcomes, as in pregestational or gestational diabetes; 2. the probability that a fetus inherits the maternal mutation is 50% and; 3. fetal insulin secretion is a major stimulus of fetal growth. Consequently, when the fetus has not inherited the maternal mutation, maternal hyperglycemia will trigger increased fetal insulin secretion and growth, with a high risk of macrosomia. By contrast, when the fetus has inherited the maternal mutation, its insulin secretion is set at the same threshold as the mother’s, and no fetal growth excess will occur. Thus, treatment of maternal hyperglycemia is necessary only in the former situation, and will lead to a risk of fetal growth restriction in the latter. It has been recommended that the management of diabetes in GCK-MODY pregnant women should be guided by assessment of fetal growth by serial ultrasounds, and institution of insulin therapy when the abdominal circumference is ≥ 75th percentile, considered as a surrogate for the fetal genotype. This strategy has not been validated in women with in GCK-MODY. Recently, the feasibility of non-invasive fetal genotyping has been demonstrated, that will improve the care of these women. Several challenges persist, including the identification of women with *GCK*-MODY before or early in pregnancy, and the modalities of insulin therapy. Yet, retrospective observational studies have shown that fetal genotype, not maternal treatment with insulin, is the main determinant of fetal growth and of the risk of macrosomia. Thus, further studies are needed to specify the management of *GCK*-MODY pregnant women during pregnancy.

## Introduction

Heterozygous pathogenic variants of the glucokinase (*GCK*) gene are associated with an autosomal dominant monogenic diabetes called *GCK*-maturity-onset-diabetes of the young (*GCK*-MODY). The *GCK*-MODY phenotype is restricted to a mild hyperglycemia that usually does not require any treatment. However, during pregnancy, the glucose levels of *GCK*-MODY mothers are high enough to potentially generate adverse outcomes similar to those observed in other forms of pregestational or gestational diabetes. The goal of the present review is to summarize current knowledge and challenges about this condition, its diagnosis, and its treatment during pregnancy. The literature was searched using the terms “glucokinase” or “GCK-MODY” or “MODY2” and “pregnancy”, and all clinical publications were reviewed. Single case reports were not retained, unless they provided important information (e.g. the occurrence of congenital malformations).

## Diabetes in Pregnancy

The relationships between diabetes and pregnancy are classically considered, according to the onset of diabetes before the beginning of pregnancy (“pregestational diabetes”) or during the course of the pregnancy (“gestational diabetes”) (1). Pregestational diabetes may be responsible for complications such as miscarriage, the occurrence of congenital malformations, and the subsequent increased risk of fetal or neonatal mortality. These complications are mainly related to the degree of hyperglycemia during the very first weeks of pregnancy (2). Pregestational as well as gestational diabetes may be responsible for many adverse issues for the mother, such as an increased frequency of hypertension, pre-eclampsia, pre-term delivery, and cesarean delivery; and for the baby, particularly macrosomia (i.e. excessive growth for gestational age), the associated risks of dystocia and neonatal hypoglycemia, and the consequences of prematurity (1). Moreover, maternal hyperglycemia might be responsible for the long-term occurrence of obesity and metabolic or cardiovascular diseases in the exposed offspring (3–6), although this remains a matter of debate (7).

The deleterious role of hyperglycemia during pregnancy has been further demonstrated by intervention studies in which “near normal” maternal blood glucose levels led to a decrease of adverse events (8, 9). These observations have led to a “glucocentric” approach of diabetes care in pregnancy, that is too restrictive (10), and other potentially modifiable risk factors have been identified, such as pre-pregnancy obesity, excessive weight gain during pregnancy, and gestational hypertension (11–13). Recent studies also showed that in women with gestational diabetes, beyond the degree of hyperglycemia, the respective roles of insulin deficiency and insulin resistance in the pathophysiology of mother’s diabetes may have differential impacts on pregnancy adverse events (14, 15). Moreover, in addition to maternal blood glucose levels, the fetal genotype may strongly influence birthweight and the risk of macrosomia (16).

## Monogenic Diabetes due to Glucokinase Molecular Alterations

Monogenic diabetes (MgD) due to pathogenic alterations of the glucokinase gene (*GCK*), the first identified genetic subtype of MODY (17), called *GCK*-MODY (formerly MODY2), is among the most common MgD subtype (18), with an estimated prevalence of 0.1% in the general population (19). Its unique characteristics have allowed drawing important lessons, particularly on the role of maternal and fetal genetics in the consequences of diabetes during pregnancy.

### The Genetics of *GCK*-MODY

Heterozygous inactivating variants in *GCK* are responsible for *GCK*-MODY. More than 900 *GCK* mutations have been reported (Human Gene Mutation Database, HGMD-2021-3*)*, including single-nucleotide variants (SNV) and rare exonic or gene deletions (20). The variants are distributed throughout the gene, with no mutational hotspots, and most are private. Pathogenic *GCK* variants lead to altered enzyme kinetics and more rarely to enzyme instability (20). No genotype/phenotype correlations have been reported (18, 21), with a similar phenotype in most patients, probably due to partial compensation of glucokinase activity by the wild-type allele (22). The molecular diagnosis of *GCK*-MODY in a proband relies on the search of SNV and large deletions, based on either analysis restricted to *GCK* (including sequencing and dosage analysis) if the proband’s phenotype is highly suggestive of *GCK*-MODY, or the sequencing of a multigene panel including *GCK*. In all cases, determining whether an identified variant is disease-causing, a normal variation, or a variant of unknown significance is a key step in the diagnostic process reviewed in (23).

### Pathophysiology of *GCK*-MODY

Glucokinase catalyzes the phosphorylation of glucose to glucose-6-phosphate, the first and rate-limiting step of glucose metabolism in the pancreatic beta-cell, which regulates insulin secretion in proportion to glucose metabolism within the physiological range. Glucokinase is thus considered as the “glucose sensor” of the pancreas (24). In patients with *GCK*-MODY, the curve of insulin secretion in response to increasing glucose concentrations is shifted to the right, and the glucose threshold for insulin release is higher than in normal individuals (25). Thus, fasting hyperglycemia is one main metabolic alteration in *GCK*-MODY. In the liver, glucokinase catalyzes the first step of glucose storage by glycogen synthesis, and patients with *GCK*-MODY harbor a decrease of hepatic glycogen synthesis and a relative increase of neoglucogenesis, which both participate to increased post-prandial glucose levels (26). *GCK* is also expressed in the pancreatic alpha-cells and in hypothalamus, and counterregulation to hypoglycemia also occurs at higher blood glucose levels in *GCK*-MODY patients (27, 28).

Insulin sensitivity is usually considered to be unaffected in *GCK*-MODY patients. However, studies showed that it was lower in patients with *GCK* mutations as compared to their non-affected relatives, and was negatively associated with a mild deterioration of glucose tolerance, consistent with a deleterious effect of chronic hyperglycemia (29–31). Conversely, a longitudinal study suggested that the deterioration of glucose tolerance observed in some *GCK*-MODY patients was due to a decrease in insulin sensitivity, which could be related to aging, weight gain, and/or polygenic susceptibility (32). Whether this may also occur during pregnancy, a known situation of decreased insulin sensitivity, is not known. To our knowledge, no longitudinal study assessed whether glucose tolerance deteriorates in women with *GCK*-MODY during pregnancy, and improves after delivery. However, the fasting and 2 hours after a 75 g oral glucose tolerance test (OGTT) blood glucose values measured in 44 pregnant women with *GCK*-MODY were in the same range as those of non-pregnant *GCK*-MODY patients (19).

### The Main Characteristics of *GCK*-MODY Patients

As compared to Type 1, Type 2 and other monogenic diabetes, *GCK*-MODY has unique characteristics reviewed in (18). The main feature observed in *GCK*-MODY patients is a mild fasting hyperglycemia, typically in a narrow range (5.4-8.3 mmol/L), with an increase 2 hours after a 75 g OGTT usually < 3.0 mmol/L (21, 33, 34). HbA_1c_ values are comprised between 38 and 60 mmol/mol (5.6-7.6%), and allow good discrimination of the patients from non-carriers relatives, and from patients with Type 1 or Type 2 diabetes (35). Hyperglycemia is present from birth and blood glucose levels increase mildly with age, as observed in non-diabetic individuals, albeit at a higher level (33, 35, 36). The penetrance of this phenotype is complete, all carriers of a *GCK* defect being hyperglycemic, generally at the same level (33).

Patients with *GCK*-MODY are clinically asymptomatic and, in the absence of other risk factors, micro- and macrovascular complications are rare, except for a mild non-clinically significant retinopathy, even in patients with long-standing hyperglycemia (36).

Consequently, outside pregnancy, no treatment of hyperglycemia is warranted in *GCK*-MODY patients. Moreover, treatments of hyperglycemia are not effective in these patients. In prospective studies, 20-50% of *GCK*-MODY patients were treated with oral hypoglycemic agents or insulin before the diagnosis was made. HbA_1c_ values were very similar in treated and untreated patients, and were not affected by treatment withdrawal (37–39). It is likely that the up-regulated counterregulation of *GCK*-MODY patients will prevent strict normalization of blood glucose values (27, 28).

## Risks Associated With Pregnancy in Women With *GCK*-MODY

### The Rate of Miscarriage Would Be Expected to Be Increased Given the Blood Glucose and HbA_1c_ Levels Observed in *GCK*-MODY Women

It has been reported to be increased compared to that of the general population (33% of 56 pregnancies) in one study (40), but similar to the general population rate in a further study by the same group on a larger population (17% of 119 pregnancies) (41), and in an additional independent study (19% of 128 pregnancies) (42).

### The Risk for Congenital Malformations Has Not Been Systematically Assessed

Blood glucose levels of *GCK*-MODY patients are compatible with a slightly increased risk, i.e. a 30% risk increase per 1% (11 mmol/mol) increase in HbA_1c_ above 6.3% (45 mmol/mol), if one refers to data obtained in women with pregestational diabetes (2). One case of caudal regression syndrome was reported, and the potential benefit of systematic peri-conceptual folic acid supplementation in women with known *GCK*-MODY has been suggested (as actually recommended in the general population) (43). A pulmonary valve stenosis was reported in a child born to a *GCK*-MODY mother, whose early pregnancy HbA_1c_ was 6.5% (48 mmol/mol) (44). Also, 4 congenital malformations were reported among 99 offspring born to mothers with *GCK*-MODY, which could be higher than in the general population, but no further information was available (41). Thus, in *GCK*-MODY women with a pre-conceptual HbA_1c_ > 6.3% (45 mmol/mol), insulin therapy could be recommended to prevent the risk of congenital malformations.

### Unaffected Offspring of Mothers With *GCK*-MODY Are at High Risk of Macrosomia

Blood glucose and HbA_1c_ values observed in patients with *GCK*-MODY confer a high risk of macrosomia (45). In women with pregestational diabetes, the probability of large for gestational age (LGA, defined by a birthweight > 90^th^ percentile) offspring increases linearly with third trimester HbA_1c_ values above 36.6 mol/mol (5.5%) and is almost maximal (65% risk) for a 53 mmol/mol (7.0%) value (12). Even in offspring born to women with mild gestational diabetes, the risk of LGA increased linearly with increasing blood glucose levels up to 5.8 mmol/L fasting and to 11.1 mmol/L at 2 hours of a 75 g OGTT, and was associated with a parallel increase in cord blood C-peptide levels (a measure of insulin secretion by the fetus) (46).

Given the autosomal transmission of *GCK*-MODY, at each pregnancy the probability that a fetus will inherit the maternal *GCK* mutation is 50%. In this context, fetal growth will dependent both on the degree of maternal hyperglycemia, and on the ability of the fetus to increase insulin secretion in response to hyperglycemia, i.e., its *GCK* genotype. Indeed, the seminal report by A.T. Hattersley et al. on *GCK*-MODY families clearly showed that non-affected offspring born to affected mothers had a 600 g higher birth weight than affected offspring born to affected mothers and than non-affected offspring born to non-affected mothers (47). According to the Pedersen hypothesis (48), this suggested that accelerated fetal growth was induced by increased fetal insulin secretion by non-affected fetuses in response to maternal hyperglycemia, and that fetal growth was normal in affected fetuses since their insulin secretion was set at the same level as their mother’s. Observational studies have consistently confirmed this report (41, 42, 49–52), and showed that the frequency of macrosomia (birthweight > 4000 g) and/or of LGA offspring were high (33-65%) in the first situation, as compared to the latter (4-13%, i.e. the expected rate of LGA in the general population) (**Table 1**). However, in one series 5/15 affected offspring born to affected mothers were macrosomic. These five infants were born to 4 mothers who had had 10 other pregnancies, and delivered 8 macrosomic infants. This suggested that confounding factors, yet unidentified, may have played a role in the occurrence of excessive fetal growth (42). Of note, in the offspring of an unaffected mother inheriting a *GCK* mutation from their father, birth weight was reduced by 500 g, compared to controls, confirming the central role of fetal insulin secretion in the fetal growth (47).

**Table 1:** Birthweight percentiles, frequency of large for gestational age newborns and gestational age at delivery according to fetal genotype and treatment of diabetes in GCK-MODY mothers.

Reference N°	Effect of fetal genotype		Effect of fetal genotype and maternal treatment			
	GCK –	GCK +	GCK – diet	GCK – insulin	GCK + diet	GCK + insulin
(50)	Pc = 85 ± 21 (38)	Pc = 47 ± 31 (44)*	Pc = 86 ± 22 (19)	Pc = 84 ± 21 (19)	Pc = 51 ± 30 (30)	Pc = 39 ± 33 (14)
	LGA = 21/38 (55%)	LGA = 4/44 (9%)*	–	–	–	–
	T = 38.1 ± 1.7	T = 38.7 ± 2.6^†^	T = 38.9 ± 1.7	T = 37.3 ± 1.1^‡^	T = 39.1 ± 2.7	T = 37.8 ± 2.0^‡^
(51)	–	–	–	–	–	–
	LGA = 9/22 (41%)	LGA = 4/45 (9%)*	–	–	–	–
	T = 38.7 ± 2.7	T = 39.3 ± 2.3	–	–	–	–
(52)	Pc = 75 ± 27 (12)	Pc = 41 ± 31 (28)*	Pc = 86 ± 10 (8)	Pc = 53 ± 37 (4)^‡^	Pc = 41 ± 31 (19)	Pc = 40 ± 31 (9)
	LGA = 4/12 (33%)	LGA = 1/28 (4%)*	–	–	–	–
	T = 39.3 ± 1.0	T = 38.4 ± 2.3	T = 39.3	T = 39.4	T = 38.8	T 37.6
(42)	–	–	Pc = 90 ± 8 (3)	Pc = 84 ± 22 (9)	Pc = 58 ± 33 (15)	Pc = 34 ± 27 (8)
	–	–	–	–	–	–
	–	–	T = 36	T = 37	T = 40.4	T = 38.0^‡^
(41)	–	–	Pc = 69 ± 34 (12)	Pc = 92 ± 18 (11)	Pc = 50 ± 28 (28)	Pc = 64 ± 35 (11)
	LGA = 15/23 (65%)	LGA = 5/39 (13%)*	LGA = 6/12 (50%)	LGA = 9/11 (82%)	LGA = 1/28 (4%)	LGA = 4/11 (36%)
	–	–	T = 39.5 ± 1.5	T = 38.3 ± 1.0^‡^	T = 39.6 ± 1.0	T = 38.7 ± 1.4^‡^

Data are: 1^st^ line: mean ± SD of birth weight percentiles (Pc) with numbers of cases into parentheses; 2^nd^ line: numbers of large for gestational age (LGA) newborns/total numbers of newborns, with percentages into parentheses; and 3^rd^ line: mean term (T) at delivery (weeks). LGA was defined as a corrected birthweight > 90^th^ percentile. *significantly lower than in GCK - babies; ^†^significantly higher than in GCK - babies; ^‡^significantly lower than in diet treated babies.

Macrosomia can be associated with increased frequencies of many perinatal adverse outcomes, including shoulder dystocia, fetal distress, perineal tears, induced preterm delivery and Cesarean delivery (CS), neonatal hypoglycemia, and neonatal respiratory distress. These complications have been reported in the context of pregestational diabetes, as well as in mild gestational diabetes, where blood glucose levels are quite similar to that observed in *GCK*-MODY (46). Shoulder dystocia was reported in 4/15 macrosomic non-affected babies born to *GCK*-MODY mothers (50). Other adverse outcomes, mainly emergency or planned CS due to macrosomia, have also been reported (42). However, the rates of these complications have not been systematically assessed in unaffected offspring born to affected mothers, compared to affected offspring.

### Unaffected Offspring of Mothers With *GCK*-MODY Do Not Exhibit Clinical or Metabolic Abnormalities in the Long Term

In various situations, fetal exposure to maternal diabetes has been associated with long-term deleterious effects, particularly defects in glucose-stimulated insulin secretion and hyperglycemia. This has been shown in offspring of mothers with type 2 diabetes (53–55), with monogenic diabetes associated with hepatocyte nuclear factor 1 alpha (56), but also with type 1 diabetes (57). As regards *GCK*-MODY, two studies demonstrated no obvious long-term effects in offspring of *GCK*-MODY mothers. In the first one, 42 adult non-mutation carriers born to *GCK*-MODY mothers were compared to 39 non-mutation carriers born to unaffected mothers, at a median age of 42 and 36 years, respectively. No differences were observed in fasting and post-load (75 g OGTT) glucose values, insulin secretion and insulin sensitivity indexes, body mass index (BMI), blood pressure and lipid profiles (49). In the second study, 15 unaffected offspring of *GCK*-MODY mothers were compared to 14 unaffected offspring of *GCK*-MODY fathers. Although the former were on average 720 g heavier at birth, they did not display any alteration of glucose tolerance, insulin secretion, BMI, percentage of body fat mass, and blood pressure, at ~ 36-39 years of age (58). These observations suggest that fetal exposure to maternal hyperglycemia (“metabolic imprinting”) is not always sufficient to induce long-term metabolic abnormalities in the offspring.

## The Diagnosis of *GCK*-MODY Before and During the Course of Pregnancy

### Outside the Pregnancy, the Diagnosis of *GCK*-MODY May Be Raised on the Discovery of Hyperglycemia

In young and lean individuals, type 1 diabetes should be excluded by the absence of diabetes-related autoantibodies (59). The criteria in favor of *GCK*-MODY include the mild degree of hyperglycemia (5.5-8.0 mmol/L), a 2 hr increment < 4.6 mmol/L on a 75 g OGTT, and a family history of hyperglycemia, including gestational diabetes, suggesting an autosomal dominant inheritance (34). A young age and a normal BMI at the time of first recognition of hyperglycemia are also in favor of the diagnosis, since they are not typical of Type 2 diabetes. The family history of hyperglycemia may be unrecognized, or even absent in the rare cases of *de novo* occurrence of a *GCK* mutation. In all cases, the suspicion of *GCK*-MODY in a proband should lead to measure fasting blood glucose in the parents (34). It is also important to systematically screen fasting blood glucose in relatives of a patient with *GCK*-MODY, since this will identify women of child-bearing age who should be genetically tested. Nevertheless, it has been estimated that almost all *GCK*-MODY cases are not diagnosed (19). Thus, it can be anticipated that in the majority of women with *GCK*-MODY hyperglycemia will be first detected during pregnancy, owing to screening for gestational diabetes.

### Screening for Gestational Diabetes Is an Opportunity to Diagnose *GCK*-MODY

For decades, the diagnosis of gestational diabetes has been a matter of debate as regards the women who should be screened, the optimal term of pregnancy to perform screening, and which test should be used (60). Some have suggested to screen all women when planning pregnancy, or at the latest at first prenatal contact, by measuring fasting plasma glucose (61, 62), which could be a good opportunity for *GCK*-MODY diagnosis. However, it is generally recommended to screen women with risk factors (age ≥ 35 years, pre-pregnancy BMI ≥ 25 kg/m², first-degree relative with diabetes, history of gestational diabetes, or of delivery of a macrosomic neonate), as soon as possible during pregnancy, and all women at 24 weeks of gestation (60). Among the risk factors for gestational diabetes, some may be present in women with *GCK*-MODY.

The reported prevalence of *GCK*-MODY among women with a diagnosis of gestational diabetes is typically 0.5-2%, but varies considerably (from 0 to 80%) according to the criteria used for genetic screening reviewed in (63, 64). Using stringent criteria to select women for genetic screening will increase the diagnosis rate, but at the cost of a lower sensitivity. New pregnancy-specific screening criteria (NSC) have been defined in a population-based study of women with gestational diabetes, of whom ~ 1% had a confirmed *GCK*-MODY (19). They include an antepartum fasting blood glucose ≥ 5.5 mmol/L (99 mg/dl) and a pre-pregnancy BMI < 25 kg/m². In the studied white European population, these criteria had a 68% sensitivity and a 99% specificity for the diagnosis of *GCK*-MODY. Using these criteria, the number of women needed to test to find one *GCK*-MODY case was 2.7. Decreasing or increasing the BMI threshold decreased or increased sensitivity, respectively, with no major effect on specificity (19). A multiethnic Australian study confirmed that these criteria performed well in women of Anglo-Celtic origin, but not in those of Asian or Indian origin (65). Among Danish women with diet-treated gestational diabetes, 2% had *GCK*-MODY, but a BMI < 25 kg/m² was not discriminant in this study (66). Thus, more multiethnic studies in women with gestational diabetes are needed to refine the criteria for genetic screening, and the diagnosis of *GCK*-MODY in early pregnancy is currently an unsolved challenge.

## Management of Pregnancy in Women With *GCK*-MODY

### Current Recommendations on the Treatment of *GCK*-MODY During Pregnancy

These recommendations mainly apply to the use of insulin therapy to prevent macrosomia and its potential consequences reviewed in (67). As mentioned, when the fetus has not inherited the maternal mutation, the risk of macrosomia is high, and normalization of maternal blood glucose levels is necessary. By contrast, when the fetus has inherited the maternal mutation, treatment of maternal hyperglycemia should be avoided because of the risk of fetal growth restriction due to a decrease of fetal blood glucose values under the insulin secretion threshold (68, 69). Thus, knowing the fetal genotype will determine whether the maternal hyperglycemia should be treated. The proof of concept of this approach was obtained in two *GCK*-MODY women in whom chorionic villous sampling, performed for other reasons, showed the presence of the mutation in the fetuses. No treatment of diabetes was initiated and the women delivered normal-weight babies (70, 71).

However, invasive prenatal diagnosis conveys risks and is not appropriate for a benign condition, and the fetal genotype is unknown in almost all cases. Thus, so far it has been suggested that the course of fetal growth, monitored by serial ultrasounds (US), may be used as a surrogate for the fetal genotype, and for the need for treatment of maternal hyperglycemia (18).

This strategy is adapted from studies performed in women with “common” gestational diabetes. All offspring of women with gestational diabetes are not at risk for macrosomia, and “too tight” control of maternal hyperglycemia may lead to an increased risk of intrauterine growth retardation (72). Thus, several randomized trials were performed in women with gestational diabetes, comparing the initiation of insulin therapy based on maternal blood glucose values, or on an accelerated fetal growth, defined by an abdominal circumference (AC) ≥ 70^th^-75^th^ percentile on US (73–76). US allowed to identify the infants at low risk of macrosomia, and to avoid insulin therapy in their mothers, with no increase of pregnancy adverse outcomes, particularly no increase of LGA, nor of small for gestational age offspring (77). Of note, insulin therapy was also introduced in the US groups when maternal blood glucose values exceeded safety levels, 6.7 mmol/L (120 mg/dl) fasting or 11.1 mmol/L (200 mg/dl) post-meal, i.e., much above the usually recommended targets in women with gestational diabetes. However, when insulin therapy had to be initiated in at risk pregnancies, strict blood glucose targets (4.4 and 6.1 mmol/L, 80 and 110 mg/dl, fasting and after meals, respectively) were set to reduce the risk of macrosomia (77). Although this approach has been validated in a real life setting (78), its benefits have been questioned in a recent review (79) and it is not part of the current guidelines on the management of gestational diabetes (1).

**Figure 1:**
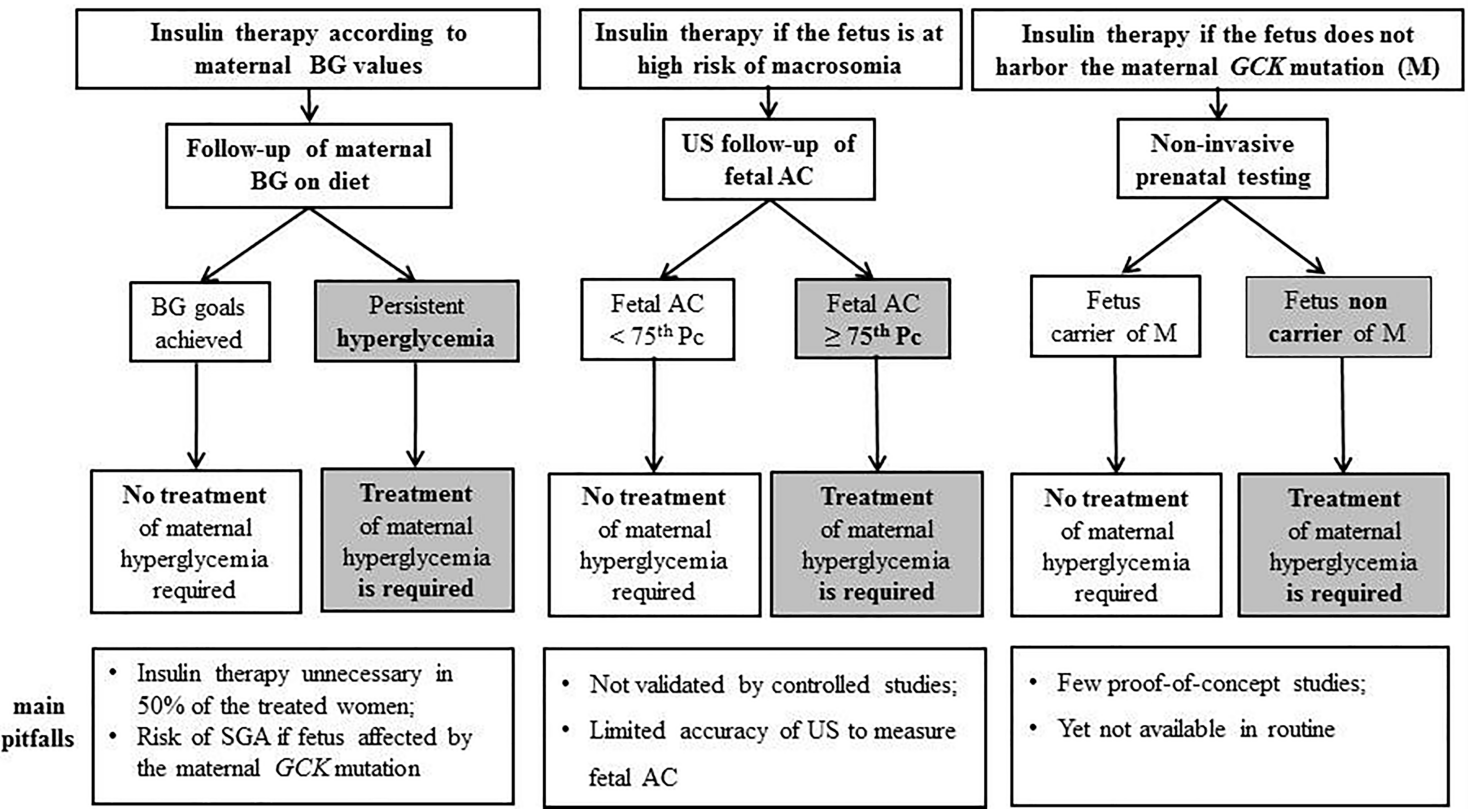
Suggested algorithms to initiate insulin therapy in pregnant women with *GCK*-MODY. The left part of the figure describes the approach based, as in “common” gestational diabetes, on maternal blood glucose (BG) values. The middle part illustrates current recommendations, based on the serial measurement of fetal abdominal circumference (AC) by ultrasounds (US), and initiation of insulin therapy when AC is ≥ 75^th^ percentile (Pc), which suggests the absence of the maternal *GCK* mutation in the fetus and a risk of macrosomia. In the right part, initiation of insulin therapy will be based on the absence of the maternal mutation (M) in the fetus, diagnosed by non-invasive prenatal testing. The bottom part of the figure indicates the main pitfalls of each strategy. SGA, small for gestational age.

The same approach has been recommended in pregnant women with *GCK*-MODY (68), including an US every two weeks, starting from 26 weeks of gestation, with the AC ≥ 75^th^ pc threshold for initiation of insulin therapy (**Figure 1**). In women treated with insulin, it is recommended that delivery should be induced at 38 weeks (18). Several potential pitfalls can be raised, including the limited accuracy of fetal US to predict the risk of macrosomia, due to intra-and inter-observer variability of AC measurement, the restricted availability of high-quality US, and the relatively late identification of the risk of macrosomia, at a time when intervention might be less efficient (80). To date, this strategy has not been validated in *GCK*-MODY and should be considered as expert opinion.

A prospective study has been recently completed in women with *GCK*-MODY, comparing the two strategies, i.e., institution of insulin therapy according to blood glucose values or to fetal growth (ClinicalTrials-NCT02556840). Analysis is ongoing and will hopefully show whether the second approach is safe, and to what extent insulin therapy is efficient to control maternal blood glucose levels and fetal growth in unaffected offspring.

### Non-Invasive Prenatal Testing

The presence of fetal DNA in maternal plasma from the early first trimester of gestation has allowed the development of non-invasive prenatal testing (NIPT) for single-gene diseases (81). However, NIPT for maternally inherited variants presents technological and analytical challenges because only a small proportion (5% to 20%) of the total cell-free DNA in maternal plasma is derived from the fetus during early pregnancy. Two major methods for NIPT have been developed. One uses droplet digital PCR to quantify reference and alternate alleles and to estimate the allelic balance of the mutation itself. The second consists in the identification of at-risk maternal haplotype at a specific locus using high-throughput DNA sequencing technologies and the determination of the maternal haplotype transmitted to the fetus, based on relative haplotype dosage estimation. Both methods have recently been successfully performed in pregnant women with *GCK*-MODY as proof-of-concept studies (82–84). In these studies, NIPT could effectively be performed from 12 weeks of gestation, with a current 3-5 week time to results, and both high sensitivity (87%) and specificity (100%) (82). The possibility of an early diagnosis could also improve the efficiency of treatment of maternal hyperglycemia to prevent macrosomia (80). Yet, NIPT is not routinely available for *GCK*-MODY but these preliminary results are promising, particularly those based on relative haplotype dosage estimation. This latter approach will benefit from the development of novel high-throughput sequencing technologies based on long read sequencing, facilitating the reconstruction of haplotypes.

### Is Insulin Therapy Effective in Women With *GCK*-MODY?

Although it is currently recommended to institute insulin therapy in *GCK*-MODY women whose babies are at high risk of macrosomia, this strategy has not been implemented in routine practice (41). Thus, in the majority of reported cases, insulin therapy was instituted on the basis of maternal capillary blood glucose upon diet, as recommended in “common” gestational diabetes, or on the discovery of macrosomia by routine US (41) (**Figure 1**). Moreover, no prospective study as defined the best modalities of the treatment and, outside pregnancy, no effect of insulin therapy on HbA_1c_ levels was observed (37–39).

In pregnant women with *GCK*-MODY, scarce reports suggested that insulin therapy may be effective. During two consecutive pregnancies, one woman was treated with insulin, 1 U/kg/d from 10-12 gestation weeks, with normalization of fructosamine values. She delivered a small-for-gestational age (1^st^ percentile) unaffected baby, and a normal weight (30^th^ percentile) affected baby (68). In another report, one woman treated with 1.43 U/kg/d at 30-38 gestation weeks delivered a normal weight (25^th^ percentile) unaffected baby (83). Also, a Japanese study reported that the mean birth weight was lower in unaffected babies born to insulin-treated vs. diet treated mothers [(52), **Table 1**].

However, several retrospective studies have assessed the respective effects of fetal genotype and of treatment with diet or insulin on pregnancy outcomes, and showed that the main determinant of offspring birth weight and of the risk of macrosomia was the fetal genotype, not the treatment of the mother (**Table 1**). Specifically, birth weights were higher in non-affected vs. affected babies, irrespective of the treatment (insulin vs. diet) (41, 42, 50–52). In all studies but one, treatment with insulin did not significantly lower birth weight of affected or unaffected offspring (**Table 1**). In one study, LGA (51 vs. 26%) and neonatal hypoglycemia (24 vs. 3%) were even more frequent in offspring of insulin-treated vs. diet-treated mothers (41). Insulin therapy was also associated with undesirable side effects. The occurrence of maternal hypoglycemia in 56% of the women, including severe hypoglycemia in 23%, was reported in one series (42). Moreover, in almost all studies, insulin therapy was associated with lower gestational age at birth, and with a higher incidence of labor induction and Cesarean deliveries, likely reflecting obstetricians’ concerns when pregnant women are treated with insulin (50).

Several hypotheses have been made to explain the poor results of insulin therapy in this context. All these studies were retrospective and some spanned over several decades. Selection bias likely occurred, leading to treat more frequently women with a more pronounced hyperglycemia and/or with already large babies, as suggested by the higher rate of macrosomia in offspring born to insulin-treated mothers in one study (41). Also, insulin dosage was highly variable, ranging 0.1-1.5 U/kg/d, and often may have been insufficient to lower blood glucose levels to the strict targets required to prevent accelerated fetal growth (85). Indeed, one series reported fasting and post-meal blood glucose profiles, recorded in 16 insulin-treated mothers with *GCK*-MODY during the first and the third trimester of pregnancy, that demonstrated glucose values well above recommended targets in almost all women (41). Gestational age at initiation of insulin therapy was also highly variable (from pre-conceptual to 38 weeks of gestation), while it has been suggested that early normalization of maternal blood glucose is necessary to prevent macrosomia reviewed in (80). Lastly, one further barrier could be the up-regulated counterregulation in *GCK*-MODY that will prevent strict normalization of blood glucose values. It has been suggested that high insulin doses, e.g. ≥ 1 U/kg/d., may be needed to normalize blood glucose, but this may be at the cost of a high risk of hypoglycemia. These difficulties are well recognized and account for the recommendation to induce delivery at 38 weeks of gestation in *GCK*-MODY women treated with insulin (18, 50).

## In Several Areas There Could Be Opportunities to Improve the Management and the Prognosis of Pregnancy in Women With *GCK*-MODY

### Improving the Diagnosis of *GCK*-MODY in the Women Before and During Pregnancy

Since patients are asymptomatic the diagnosis of *GCK*-MODY relies on systematic screening. Information about monogenic diabetes should be delivered to healthcare workers and in the general population. The fortuitous discovery of a mild hyperglycemia should not be neglected. First-degree relatives of probands with *GCK*-MODY should systematically be screened.

During pregnancy, early diagnosis of *GCK*-MODY is difficult since currently only women with risk factors for gestational diabetes are screened in the first trimester. Also, the performance of algorithms to select hyperglycemic women to be genetically screened should be evaluated prospectively in multiethnic studies.

### Improving the Diagnosis in the Fetus

In pregnant women with a confirmed *GCK*-MODY, non-invasive fetal genotyping will hopefully replace the US-guided management when routinely available. This will considerably lighten the care and the follow-up of pregnancy in women whose fetuses have inherited the mutation (**Figure 1**).

### The Best Modalities and the Efficiency of Insulin Therapy Remain to be Determined

As fetal growth is highly sensitive to mild degrees of hyperglycemia, treatment of maternal hyperglycemia, at the earliest during pregnancy and with strict glycemic targets, is mandatory to decrease adverse events (86). However, decreasing blood glucose values with insulin is associated with a high risk of maternal hypoglycemia, including severe episodes (87). Continuous glucose monitoring during pregnancy improves maternal glycemia and pregnancy outcomes, and is now recommended in pregnant women with type 1 diabetes (88). Moreover, it has been suggested that closed-loop and sensor-augmented pump insulin delivery could be efficient to lower blood glucose levels without increasing the risk of severe hypoglycemia in pregnant women (89). Whether this could be used to overcome the up-regulated counterregulation without increasing the risk of hypoglycemia in women with *GCK*-MODY is not known. Since it is difficult to perform studies in pregnant women, one may suggest to first test the feasibility of this approach in patients with *GCK*-MODY outside pregnancy.

Yet, it is unlikely that other treatments could be used. Sulfonylureas are not a good choice, since they cross the placental barrier, stimulate fetal insulin secretion and are responsible for macrosomia and neonatal hypoglycemia (90). Theoretically, metformin, alone or in association with insulin, could be used. However, metformin crosses the placental barrier, and concerns have been raised about undesirable long-term effects in exposed children (91). Moreover, its potential benefit has not been assessed in patients with *GCK*-MODY outside pregnancy.

## Conclusion

*GCK*-MODY is a quasi-experimental human model that allowed to define the respective roles of maternal hyperglycemia and fetal genotype on fetal growth, and to confirm the central role of fetal insulin secretion in fetal growth. It is also a unique situation suggesting that fetal exposure to maternal hyperglycemia is not always responsible *per se* for late adverse consequences in the offspring. Non-invasive fetal genotyping is a major advance in the care of *GCK*-MODY women, since it will allow determining those women whose diabetes should be treated during pregnancy. Challenges persist in the accurate identification of women with *GCK*-MODY before or in early pregnancy, and in the definition of therapeutic modalities during pregnancy. Multicenter studies or registers could be useful to improve our knowledge in these fields.

## Author Contributions

JT, CS-M, and CB-C wrote the draft of the manuscript. All authors contributed to the writing and the reviewing of the manuscript. All authors approved the final version of the manuscript.

## Conflict of Interest

The authors declare that the research was conducted in the absence of any commercial or financial relationships that could be construed as a potential conflict of interest.

## Publisher’s Note

All claims expressed in this article are solely those of the authors and do not necessarily represent those of their affiliated organizations, or those of the publisher, the editors and the reviewers. Any product that may be evaluated in this article, or claim that may be made by its manufacturer, is not guaranteed or endorsed by the publisher.
